# Supplementary material for: Divergence of cortical force-generating mechanisms underlies differences in spindle behavior between C. elegans and C. inopinata
Source: PLoS One. 2026 Oct 27;21(10):e0352057. doi: 10.1371/journal.pone.0352057 (PMC13712525; doi:10.1371/journal.pone.0352057)
Supplement: Supplementary file 1 [file pone.0352057.s007.pdf]

Manuscript#: PONE-D-26-27765

"Divergence of Cortical Force-Generating Mechanisms Underlies Differences in Spindle Behavior between *C. elegans* and *C. inopinata*"

Shun Oomur[1], Koji Kyoda, Shuichi Onami, Nami Haruta, Asako Sugimoto

A point-by-point response to the reviewers' comments

Reviewer 1

Reviewer comment

The conclusion that differences in the contribution of different microtubule regulators can account for the differences in pronuclear and spindle dynamics rests entirely on the use of RNAi to reduce gene function. As RNAi is known to differ in effectiveness for knocking down gene function for different genes in *C. elegans*, the authors cannot exclude the possibilities that (i) knocking down the force generators would have potentially even more severe impacts on *C. inopinata*, and more significantly (ii) knockdown of KLP-7 in *C. inopinata* might be weak relative to the knockdown in *C. elegans*, and hence account for the lack of an effect in *C. inopinata*. While it would be interesting and potentially much more conclusive to compare the impact of klp-7 null alleles in both species, the authors need to at least acknowledge this caveat concerning the variable effectiveness of RNAi and their conclusions as the different relative contributions of KLP-7 and the other microtubule regulators in the two species.

Response

We agree with the reviewer that variability in RNAi efficiency is an important consideration, particularly when comparing gene function between *C. elegans* and *C. inopinata*. Because of the technical difficulty of obtaining sufficient quantities of specific stages of early *C. inopinata* embryos for quantitative analyses such as qPCR, we were unable to directly measure and compare the extent of gene depletion between the two species. Moreover, because genome-editing methods have not yet been established in *C. inopinata*, it is currently not feasible to generate and analyze *klp-7* null mutants in this species. We therefore cannot exclude the possibility that differences in RNAi efficiency contributed to the observed differences in pronuclear and spindle dynamics, including the relatively mild effect of *klp-7(RNAi)* in *C. inopinata*.

Nevertheless, RNAi targeting *gpr-1/2*, *let-99*, or *klp-7* caused high embryonic lethality in *C. inopinata*, at levels comparable to those observed in *C. elegans*. These results indicate that the RNAi treatments produced biologically substantial reductions in gene function, although they do not establish that the extent of depletion was equivalent between the two species. Thus, while differences in RNAi efficiency may have contributed to the observed phenotypic differences, they are unlikely to account fully for all of our findings.

Following the reviewer’s suggestion, we have revised the Results and Discussion sections (Page 14, line 357; Pages 16–17, lines 418–431) to explicitly acknowledge the technical limitations of RNAi analysis in

C. inopinata and to qualify our conclusion regarding the differential contributions of KLP-7 and other microtubule regulators in the two species.

Reviewer #2:

Major points 1

Pronuclear and spindle dynamics in both species were assessed at 25°C, which near the upper limit of what *C. elegans* tolerates and may be near the lower end of what *C. inopinata* normally experiences. While the rationale for comparing the two species at the same temperature is reasonable, the report would be strengthened by evidence that the observations made for *C. inopinata* at 25°C are similar to what is seen under culture conditions closer to the preferred temperature for this species.

Response

We thank the reviewer for raising this important point regarding the temperature conditions used for the interspecies comparison. Prior to conducting this experiment, we measured the hatching rates of *C. elegans* and *C. inopinata* over a broad temperature range. More than 80% of *C. elegans* embryos hatched at temperatures between 10°C and 28°C, whereas more than 80% of *C. inopinata* embryos hatched between 16°C and 32°C. These results indicate that 25°C lies well within the permissive range for embryonic development in both species and is not close to the lower limit for C. *inopinata*.

Although these hatching data do not directly demonstrate that all aspects of pronuclear and spindle dynamics are identical across the permissive temperature range, they support the conclusion that the dynamics observed at 25°C were examined under physiologically permissive conditions for *C. inopinata*. Moreover, the use of the same temperature for both species allowed us to compare their embryonic dynamics under equivalent experimental conditions.

To clarify this rationale, we have added the temperature-dependent hatching data to the Supporting Information and described the permissive temperature ranges in the Results section (Page 8, lines 183–184).

Major point 2

The RNAi approaches for *C. elegans* and *C. inopinata* are quite different. For *C. elegans*, L4 larvae were soaked and self-fertilized embryos were analyzed from the resulting adults. For *C. inopinata*, gravid females were fed RNAi bacteria, and embryos were analyzed from the mated progeny of these females. No comparison of RNAi efficiency, other than embryonic lethality, is provided. Without assessing the degree of depletion, it is difficult to compare the severity of the resulting phenotypes between species. This is particularly important for *Cin-klp-7,* which shows a weaker phenotype than might be expected. The authors should show that this is not simply due to incomplete

depletion. If depletion efficiency cannot be measured directly, the authors should, at minimum, acknowledge this limitation in the Results and Discussion sections.

Response

We agree that differences in the RNAi protocols and efficiencies between the two species are an important limitation, particularly when interpreting the relatively mild *Cin-klp-7(RNAi)* phenotype. As this concern overlaps with that raised by Reviewer #1, we refer the reviewer to our detailed response above. Briefly, although high embryonic lethality indicates that RNAi substantially reduced gene function, we were unable to directly compare depletion efficiency between the two species and therefore cannot exclude the possibility that incomplete or variable knockdown contributed to the observed phenotypic differences. We have revised the Results and Discussion sections accordingly (Page 14, line 357; Pages 16–17, lines 418–431).

Major point 3

The data presented in Fig. 6 should be quantified similarly to what was done in Fig. 4. While the *Cin-gpr-1* RNAi phenotype is clear, the effect of *Cin-let-99* depletion is more difficult to assess. This is particularly true for the increased amplitude of spindle oscillations reported by the authors. This difference is not obvious from the data as shown and should be quantified to support the authors' interpretations.

Response

Following the reviewer's suggestion, we have quantified the data presented in Fig. 6 and added new panels (Fig. 6C–6E) in the revised manuscript. These newly added panels present the number of rocking cycles, the angular displacement during initial rocking, and the centrosome orientation upon completion of cytokinesis, respectively.

These quantitative data demonstrate that *gpr-1/2* RNAi reduced the number of rocking cycles in both species, and *let-99* RNAi increased the rocking amplitude in both species. Notably, while *klp-7* RNAi reduced the number of rocking cycles in both species, it induced spindle misalignment at the completion of cytokinesis exclusively in *C. elegans*, but not in *C. inopinata*.

We have added these new data and descriptions into the Results section (Page 14-15, lines 358–368). In addition, to reflect these quantitative analyses, we have revised the Materials and Methods section under "Quantification of mitotic spindle oscillation" (Page 6, lines 125–131).

Major point 4

In *C. inopinata*, the NCC forms more posteriorly, then moves more anteriorly, before moving back to its final position at ~60% of A-P axis. Posterior formation of the NCC is attributed to the position of the maternal pronucleus/oocyte nucleus, as well as reduced movement of the male pronucleus. What do the authors think underlies

the subsequent over-centration (i.e. increased movement of the NCC to the anterior) in *C. inopinata*?

Response

We thank the reviewer for this insightful question. We speculate that the excessive anterior displacement of the NCC in *C. inopinata* reflects a transient imbalance between anterior-directed, microtubule length-dependent pulling forces and cortical pulling forces that normally position and stabilize the NCC near the center of the embryo. Specifically, the anterior-directed forces may initially predominate, causing the NCC to move beyond the center, after which cortical pulling forces reposition it at approximately 60% of the A–P axis. We have added this possible explanation to the Discussion section (Page 17, lines 435–438).

Minor point 1

Lines 189, 217 – 219, 291 – 292, and possibly elsewhere: Add references to original papers for *C. elegans* observations, e.g. PMID: 8625834 DOI: 10.1242/dev.122.5.1467 should be referenced for oocyte nuclear positioning in *C. elegans*.

Response

Following the reviewer's suggestion, we have added references to original papers for *C. elegans* observations throughout the manuscript.

For the oocyte nuclear positioning (Page 9, line 217):

- Goldstein, B., & Hird, S. N. Specification of the anteroposterior axis in *Caenorhabditis elegans*. *Development* 1 May 1996; 122 (5): 1467–1474. doi: https://doi.org/10.1242/dev.122.5.1467

For the NCC and mitotic spindle dynamics (Page 10, Line 338):

- Albertson, D. G. Formation of the first cleavage spindle in nematode embryos. *Developmental Biology,* Volume 101, Issue 1, 1984, Pages 61-72, ISSN 0012-1606, doi: https://doi.org/10.1016/0012-1606(84)90117-9.

For the description of the Gα-GPR-LIN-5 complex (Page 13, Line 337):

- Park, D. H., & Rose, L. S. (2008). Dynamic localization of LIN-5 and GPR-1/2 to cortical force generation domains during spindle positioning. *Developmental biology*, *315*(1), 42-54.
- Grill, S. W., Gönczy, P., Stelzer, E. H., & Hyman, A. A. (2001). Polarity controls forces governing asymmetric spindle positioning in the *Caenorhabditis elegans* embryo. *Nature*, *409*(6820), 630-633.
- Tsou, M. F. B., Hayashi, A., DeBella, L. R., McGrath, G., & Rose, L. S. (2002). LET-99 determines spindle position and is asymmetrically enriched in response to PAR polarity cues in *C. elegans* embryos.

Minor point 2

Line 62: typo/grammar, remove "the" before "intracellular dynamics"

Response

Following the reviewer's suggestion, we have revised the manuscript accordingly (Page 3, Lines 61). We have also corrected similar grammatical errors elsewhere in the manuscript (Page 3, Lines 62).

Minor point 3

Lines 82-85: Since *C. inopinata* is a relatively new model, it would be helpful to add technical details regarding culture conditions here, rather than directing readers only to previous work (i.e. ref 14). Both modifications to NGM plates and any differences in propagation due to gonochorism should be added.

Response

Following the reviewer's suggestion, we have added technical details regarding the culture conditions of *C. inopinata* to the Materials and Methods section (Page 4, Lines 84–89).

Minor point 4

Line 102 – 103: The Methods state that all imaging experiments were performed at 25℃. The authors should indicate how this temperature control was achieved.

Response

Following the reviewer's suggestion, we have added the temperature control method to the Materials and Methods section (Page 5, lines 110–112).

Minor point 5

Line 147: Were antibiotics used in RNAi plates as is typical for RNAi by feeding for *C. elegans*? If so indicate which antibiotic was used and at what final concentration. If not, it would be helpful to state this and briefly explain the rationale, especially for researchers who may wish to perform RNAi in *C. inopinata*.

Response

Yes, ampicillin (final concentration, 50 μg/mL) and tetracycline (final concentration, 10 μg/mL) were used in the RNAi plates for *C. inopinata*. Following the reviewer's suggestion, we have added this information to the Materials and Methods section (Page 7, Lines 160).

Minor point 6

Lines 147 – 151: For readers unfamiliar with *C. inopinata*, please clarify the RNAi workflow. Were the "gravid adults" used for embryo dissection and imaging mated females, whose parents, both female and male, had been RNAi treated since hatching? Similarly, for embryonic lethality tests, were the females and males used the progeny of the original RNAi-treated "gravid females"?

Response

Following the reviewer's suggestion, we have clarified the RNAi workflow in the Materials and Methods section (Page 7, lines 161–165). Briefly, untreated adult females (P0) were transferred to RNAi plates and allowed to lay eggs. The F1 males and females were continuously exposed to feeding RNAi from hatching and allowed to mate with each other. The resulting mated F1 females carrying embryos were referred to as "gravid adults." Their embryos (F2) were used for both live imaging and embryonic lethality assays.

Minor point 7

Line 165: Add to main text that the duration of the first cell cycle was measured as the time between NEBD and the completion of cytokinesis. This is currently only specified in the figure legend.

Response to minor point 7

Following the reviewer's suggestion, we have revised the main text to define the duration of the first cell division as the interval from nuclear envelope breakdown (NEBD) to the completion of cytokinesis (Page 8, lines 185–186), consistent with the figure legend.

Minor point 8

Line 182 and throughout: Replace "pronucleus" with "pronuclear", when the word is used as an adjective, as in "pronuclear dynamics". This terminology/grammar is inconsistent throughout the manuscript.

Response to minor point 8

Following the reviewer's suggestion, we have replaced "pronucleus" with "pronuclear" throughout the manuscript when used adjectivally.

Minor point 9

Lines 190 – 191 and Fig. 2C: Did the authors measure oocyte volume/size? Given the similar size of the embryos, I was surprised that the oocytes and zygote shown in Fig. 2C appear substantially larger in *C. inopinata* than in *C. elegans*.

Response

Although we did not directly measure oocyte volume, *C. inopinata* eggs are significantly larger than those of *C. elegans*, with an approximately 5.7% greater egg length (Fig. 1B). This difference may reflect a corresponding difference in oocyte size.

We also found that the scale bar in the legend for Fig. 2C was incorrectly labeled as 100 µm rather than 25 µm, which likely contributed to the apparent size discrepancy. We have corrected this error in the revised manuscript (Page 9, line 236).

Minor point 10

Line 278: The phrase "both centrosomes diffused slowly" implies diffusion of intact centrosomes. Consider revising to indicate slower, progressive centrosome disassembly, possibly by diffusion of centrosome components into the cytoplasm.

Response

Following the reviewer's suggestion, we have revised the Results section by replacing "both centrosomes diffused slowly" with "the centrosome signals gradually decreased," which more accurately describes the progressive loss of centrosomal fluorescence observed in our imaging (Page 12, line 320).

Minor point 11

Line 285: Add a description to the figure legend for Fig. 5 as to what "1.5 x enhanced" refers to.

Response

We thank the reviewer for pointing this out. The label "1.5 × enhanced" referred to an adjustment of the display range in ImageJ, from 90–700 for C. elegans to 90–500 for *C. inopinata*, which increased the apparent brightness by approximately 1.5-fold. However, because Fig. 5 shows different transgenes in the two species and is not intended for quantitative comparison of fluorescence intensity, we agreed that this label could be misleading. We have therefore removed "1.5 × enhanced" from Fig. 5 and its legend.

Comments to the Author

3. Have the authors made all data underlying the findings in their manuscript fully available?

The PLOS Data policy requires authors to make all data underlying the findings described in their manuscript fully available without restriction, with rare exception (please refer to the Data Availability Statement in the manuscript PDF file). The data should be provided as part of the manuscript or its supporting information, or deposited to a public repository. For example, in addition to summary statistics, the data points behind means, medians and variance measures should be available. If there are restrictions on publicly sharing data—e.g. participant privacy or use of data from a third party—those must be specified.

Reviewer #2: No

Response

In response to the reviewer's comment, we have provided all underlying numerical data required to replicate the findings of this study as "Supporting Information" in the public repository "Tohoku University Research Data Lake IZUMI." The corresponding URL has been incorporated into the revised manuscript (Page 23-24, lines 582–604).
