# Supplementary material for: Divergence of cortical force-generating mechanisms underlies differences in spindle behavior between C. elegans and C. inopinata
Source: PLoS One. 2026 Oct 27;21(10):e0352057. doi: 10.1371/journal.pone.0352057 (PMC13712525; doi:10.1371/journal.pone.0352057)
Supplement: Peer review history

# Peer review: Decision Letter 0

**DOI:** 10.1371/journal.pone.0352057.r001

**Authors:** Bob Goldstein

28 Jun 2026

*C. elegans*  and *C. inopinata*

Dear Dr. Sugimoto,

Please submit your revised manuscript by Aug 27 2026 11:59PM. If you will need more time than this to complete your revisions, please reply to this message or contact the journal office at plosone@plos.org. When you're ready to submit your revision, log on to https://www.editorialmanager.com/pone/ and select the 'Submissions Needing Revision' folder to locate your manuscript file.

- A letter that responds to each point raised by the academic editor and reviewer(s). You should upload this letter as a separate file labeled 'Response to Reviewers'.
- A marked-up copy of your manuscript that highlights changes made to the original version. You should upload this as a separate file labeled 'Revised Manuscript with Track Changes'.
- An unmarked version of your revised paper without tracked changes. You should upload this as a separate file labeled 'Manuscript'.

If you would like to make changes to your financial disclosure, please include your updated statement in your cover letter. Guidelines for resubmitting your figure files are available below the reviewer comments at the end of this letter.

If applicable, we recommend that you deposit your laboratory protocols in protocols.io to enhance the reproducibility of your results. Protocols.io assigns your protocol its own identifier (DOI) so that it can be cited independently in the future. For instructions see: https://journals.plos.org/plosone/s/submission-guidelines#loc-laboratory-protocols. Additionally, PLOS ONE offers an option for publishing peer-reviewed Lab Protocol articles, which describe protocols hosted on protocols.io. Read more information on sharing protocols at https://plos.org/protocols?utm_medium=editorial-email&utm_source=authorletters&utm_campaign=protocols.

As the corresponding author, your ORCID iD is verified in the submission system and will appear in the published article. PLOS supports the use of ORCID, and we encourage all coauthors to register for an ORCID iD and use it as well. Please encourage your coauthors to verify their ORCID iD within the submission system before final acceptance, as unverified ORCID iDs will not appear in the published article. *Only*  the individual author can complete the verification step; PLOS staff *cannot*  verify ORCID iDs on behalf of authors.

We look forward to receiving your revised manuscript.

Kind regards,

Bob

Bob Goldstein

Academic Editor

PLOS One

Journal Requirements:

When submitting your revision, we need you to address these additional requirements.

1. Please ensure that your manuscript meets PLOS ONE's style requirements, including those for file naming. The PLOS ONE style templates can be found at

https://journals.plos.org/plosone/s/file?id=wjVg/PLOSOne_formatting_sample_main_body.pdf and

https://journals.plos.org/plosone/s/file?id=ba62/PLOSOne_formatting_sample_title_authors_affiliations.pdf

2. Thank you for stating the following in the Acknowledgments Section of your manuscript:

"We thank members of the Sugimoto Lab for helpful discussion. Some strains were provided by the CGC, which is funded by NIH Office of Research Infrastructure Programs (P40 OD010440). This work was supported by Japan Science and Technology Agency (JST), Core Research for Evolutionary Science and Technology (CREST) Grant Number JPMJCR18S7 and Japan Society for the Promotion of Science (JSPS) KAKENHI Grant Number JP23K27152 to A.S., and JSPS KAKENHI Grant Number JP23KJ0148 and MEXT's University Fellowship Founding Project for Innovation Creation in Science and Technology to S.O."

We note that you have provided additional information within the Acknowledgements Section that is not currently declared in your Funding Statement. Please note that funding information should not appear in the Acknowledgments section or other areas of your manuscript. We will only publish funding information present in the Funding Statement section of the online submission form.

Please remove any funding-related text from the manuscript and let us know how you would like to update your Funding Statement. Currently, your Funding Statement reads as follows:

"This work was supported by Japan Science and Technology Agency (JST; https://www.jst.go.jp/), Core Research for Evolutionary Science and Technology (CREST; https://www.jst.go.jp/kisoken/crest/en/) Grant Number JPMJCR18S7 and Japan Society for the Promotion of Science (JSPS; https://www.jsps.go.jp/) KAKENHI Grant Number JP23K27152 to A.S., and JSPS KAKENHI Grant Number JP23KJ0148 and MEXT's University Fellowship Founding Project for Innovation Creation in Science and Technology (https://pgd.tohoku.ac.jp/rpc/fellowship.html) to S.O."

Please include your amended statements within your cover letter; we will change the online submission form on your behalf.

3. Thank you for stating the following financial disclosure:

“This work was supported by Japan Science and Technology Agency (JST; https://www.jst.go.jp/), Core Research for Evolutionary Science and Technology (CREST; https://www.jst.go.jp/kisoken/crest/en/) Grant Number JPMJCR18S7 and Japan Society for the Promotion of Science (JSPS; https://www.jsps.go.jp/) KAKENHI Grant Number JP23K27152 to A.S., and JSPS KAKENHI Grant Number JP23KJ0148 and MEXT's University Fellowship Founding Project for Innovation Creation in Science and Technology (https://pgd.tohoku.ac.jp/rpc/fellowship.html) to S.O.”

Please state what role the funders took in the study.  If the funders had no role, please state: "The funders had no role in study design, data collection and analysis, decision to publish, or preparation of the manuscript."

If this statement is not correct you must amend it as needed.

Please include this amended Role of Funder statement in your cover letter; we will change the online submission form on your behalf.

4. We note that your Data Availability Statement is currently as follows: "All relevant data are within the manuscript and will be provided upon request."

Please confirm at this time whether or not your submission contains all raw data required to replicate the results of your study. Authors must share the “minimal data set” for their submission. PLOS defines the minimal data set to consist of the data required to replicate all study findings reported in the article, as well as related metadata and methods (https://journals.plos.org/plosone/s/data-availability#loc-minimal-data-set-definition).

For example, authors should submit the following data:

- The values behind the means, standard deviations and other measures reported;

- The values used to build graphs;

- The points extracted from images for analysis.

Authors do not need to submit their entire data set if only a portion of the data was used in the reported study.

If your submission does not contain these data, please either upload them as Supporting Information files or deposit them to a stable, public repository and provide us with the relevant URLs, DOIs, or accession numbers. For a list of recommended repositories, please see https://journals.plos.org/plosone/s/recommended-repositories.

If there are ethical or legal restrictions on sharing a de-identified data set, please explain them in detail (e.g., data contain potentially sensitive information, data are owned by a third-party organization, etc.) and who has imposed them (e.g., an ethics committee). Please also provide contact information for a data access committee, ethics committee, or other institutional body to which data requests may be sent. If data are owned by a third party, please indicate how others may request data access.

5. If the reviewer comments include a recommendation to cite specific previously published works, please review and evaluate these publications to determine whether they are relevant and should be cited. There is no requirement to cite these works unless the editor has indicated otherwise.

Reviewers' comments:

Reviewer's Responses to Questions

**Comments to the Author**

1. Is the manuscript technically sound, and do the data support the conclusions?

Reviewer #1: Partly

Reviewer #2: Partly

**********

2. Has the statistical analysis been performed appropriately and rigorously?

Reviewer #1: Yes

Reviewer #2: Yes

**********

3. Have the authors made all data underlying the findings in their manuscript fully available?

The PLOS Data policy

Reviewer #1: Yes

Reviewer #2: No

**********

4. Is the manuscript presented in an intelligible fashion and written in standard English?

Reviewer #1: Yes

Reviewer #2: Yes

**********

Reviewer #1: Oomura et al document differerences in pronuclear positioning and migration, and mitotic spindle morphology in C. inopinata relative to C. elegans oocytes and one-cell zygotes, with C. inopinata being the one known nematode species that is most closely related to C. elegans. As these differences in cell division dynamics likely reflect differences in microtubule dynamics, the authors compare the impact of knockdowns known to influence these events in C. elegans. They find that RNAi knockdown of genes encoding proteins that influence cortical and cytoplasmic pulling forces on microtubules have substantial impacts on these dynamics in both species, while RNAi knockdown of the microtubule depolymerase KLP-7 only affects these processes in C. elegans and not in inopinata. The authors conclude that relatively minor changes in microtubule regulation can account for species differences in cell division dynamics. These are interesting results, and the comparison of dynamics in the two species is especially compelling. This manuscript would be suitable for publication in PLOS One if the authors can first address the following significant concern.

The conclusion that differences in the contribution of different microtubule regulators can account for the differences in pronuclear and spindle dynamics rests entirely on the use of RNAi to reduce gene function. As RNAi is known to differ in effectiveness for knocking down gene function for different genes in C. elegans, the authors cannot exclude the possibilities that (i) knocking down the force generators would have potentially even more severe impacts on C. inopinata, and more significantly (ii) knockdown of KLP-7 in C. inopinata might be weak relative to the knockdown in C. elegans, and hence account for the lack of an effect in C. inopinata. While it would be interesting and potentially much more conclusive to compare the impact of klp-7 null alleles in both species, the authors need to at least acknowledge this caveat concerning the variable effectiveness of RNAi and their conclusions as the different relative contributions of KLP-7 and the other microtubule regulators in the two species.

Reviewer #2: In this manuscript, Oomura et al. examine pronuclear migration and positioning of the mitotic spindle in the one-cell embryo of the closest known relative of Caenorhabditis elegans, Caenorhabditis inopinata. They find that the first mitotic division takes longer in C. inopinata, and that several events during pronuclear migration, centration and mitotic spindle assembly are different. Despite these differences, the end result – asymmetric cell division along the embryo anterior-posterior axis – is strikingly similar to C. elegans. They also compare the effect of depleting three spindle assembly or positioning factors (gpr-1, let-99 and klp-7) on embryonic lethality and spindle dynamics in C. inopinata and C. elegans. Overall, this manuscript provides an initial characterization of the first embryonic division in C. inopinata and lays the groundwork for more mechanistic comparisons of spindle assembly dynamics between these two sister species. While this work is largely well-done, there are a few places where additional experimentation would strengthen the authors’ conclusions or where the conclusions should be revised in view of current limitations.

Major points

Pronuclear and spindle dynamics in both species were assessed at 25�C, which near the upper limit of what C. elegans tolerates and may be near the lower end of what C. inopinata normally experiences. While the rationale for comparing the two species at the same temperature is reasonable, the report would be strengthened by evidence that the observations made for C. inopinata at 25°C are similar to what is seen under culture conditions closer to the preferred temperature for this species.

The RNAi approaches for C. elegans and C. inopinata are quite different. For C. elegans, L4 larvae were soaked and self-fertilized embryos were analyzed from the resulting adults. For C. inopinata, gravid females were fed RNAi bacteria, and embryos were analyzed from the mated progeny of these females. No comparison of RNAi efficiency, other than embryonic lethality, is provided. Without assessing the degree of depletion, it is difficult to compare the severity of the resulting phenotypes between species. This is particularly important for Cin-klp-7, which shows a weaker phenotype than might be expected. The authors should show that this is not simply due to incomplete depletion. If depletion efficiency cannot be measured directly, the authors should, at minimum, acknowledge this limitation in the Results and Discussion sections.

The data presented in Fig. 6 should be quantified similarly to what was done in Fig. 4. While the Cin-gpr-1 RNAi phenotype is clear, the effect of Cin-let-99 depletion is more difficult to assess. This is particularly true for the increased amplitude of spindle oscillations reported by the authors. This difference is not obvious from the data as shown and should be quantified to support the authors’ interpretations.

In C. inopinata, the NCC forms more posteriorly, then moves more anteriorly, before moving back to its final position at ~60% of A-P axis. Posterior formation of the NCC is attributed to the position of the maternal pronucleus/oocyte nucleus, as well as reduced movement of the male pronucleus. What do the authors think underlies the subsequent over-centration (i.e. increased movement of the NCC to the anterior) in C. inopinata?

Minor points

Lines 189, 217 – 219, 291 – 292, and possibly elsewhere: Add references to original papers for C. elegans observations, e.g. PMID: 8625834 DOI: 10.1242/dev.122.5.1467 should be referenced for oocyte nuclear positioning in C. elegans.

Line 62: typo/grammar, remove “the” before “intracellular dynamics”

Lines 82-85: Since C. inopinata is a relatively new model, it would be helpful to add technical details regarding culture conditions here, rather than directing readers only to previous work (i.e. ref 14). Both modifications to NGM plates and any differences in propagation due to gonochorism should be added.

Line 102 – 103: The Methods state that all imaging experiments were performed at 25C. The authors should indicate how this temperature control was achieved.

Line 147: Were antibiotics used in RNAi plates as is typical for RNAi by feeding for C. elegans? If so indicate which antibiotic was used and at what final concentration. If not, it would be helpful to state this and briefly explain the rationale, especially for researchers who may wish to perform RNAi in C. inopinata.

Lines 147 – 151: For readers unfamiliar with C. inopinata, please clarify the RNAi workflow. Were the “gravid adults” used for embryo dissection and imaging mated females, whose parents, both female and male, had been RNAi treated since hatching? Similarly, for embryonic lethality tests, were the females and males used the progeny of the original RNAi-treated “gravid females”?

Line 165: Add to main text that the duration of the first cell cycle was measured as the time between NEBD and the completion of cytokinesis. This is currently only specified in the figure legend.

Line 182 and throughout: Replace “pronucleus” with “pronuclear”, when the word is used as an adjective, as in “pronuclear dynamics”. This terminology/grammar is inconsistent throughout the manuscript.

Lines 190 – 191 and Fig. 2C: Did the authors measure oocyte volume/size? Given the similar size of the embryos, I was surprised that the oocytes and zygote shown in Fig. 2C appear substantially larger in C. inopinata than in C. elegans.

Line 278: The phrase “both centrosomes diffused slowly” implies diffusion of intact centrosomes. Consider revising to indicate slower, progressive centrosome disassembly, possibly by diffusion of centrosome components into the cytoplasm.

Line 285: Add a description to the figure legend for Fig. 5 as to what “1.5 x enhanced” refers to.

**********

what does this mean?). If published, this will include your full peer review and any attached files.

If you choose “no”, your identity will remain anonymous but your review may still be made public.

**Do you want your identity to be public for this peer review?**  For information about this choice, including consent withdrawal, please see our Privacy Policy

Reviewer #1: **Yes:**  Bruce Bowerman

Reviewer #2: No

**********

[NOTE: If reviewer comments were submitted as an attachment file, they will be attached to this email and accessible via the submission site. Please log into your account, locate the manuscript record, and check for the action link "View Attachments". If this link does not appear, there are no attachment files.]

To ensure your figures meet our technical requirements, please review our figure guidelines: https://journals.plos.org/plosone/s/figures

You may also use PLOS’s free figure tool, NAAS, to help you prepare publication quality figures: https://journals.plos.org/plosone/s/figures#loc-tools-for-figure-preparation.

NAAS will assess whether your figures meet our technical requirements by comparing each figure against our figure specifications.

# Author comment: Author response to Decision Letter 1

**DOI:** 10.1371/journal.pone.0352057.r002

17 Sep 2026

Reviewer 1

Reviewer comment

The conclusion that differences in the contribution of different microtubule regulators can account for the differences in pronuclear and spindle dynamics rests entirely on the use of RNAi to reduce gene function. As RNAi is known to differ in effectiveness for knocking down gene function for different genes in C. elegans, the authors cannot exclude the possibilities that (i) knocking down the force generators would have potentially even more severe impacts on C. inopinata, and more significantly (ii) knockdown of KLP-7 in C. inopinata might be weak relative to the knockdown in C. elegans, and hence account for the lack of an effect in C. inopinata. While it would be interesting and potentially much more conclusive to compare the impact of klp-7 null alleles in both species, the authors need to at least acknowledge this caveat concerning the variable effectiveness of RNAi and their conclusions as the different relative contributions of KLP-7 and the other microtubule regulators in the two species.

Response

We agree with the reviewer that variability in RNAi efficiency is an important consideration, particularly when comparing gene function between C. elegans and C. inopinata. Because of the technical difficulty of obtaining sufficient quantities of specific stages of early C. inopinata embryos for quantitative analyses such as qPCR, we were unable to directly measure and compare the extent of gene depletion between the two species. Moreover, because genome-editing methods have not yet been established in C. inopinata, it is currently not feasible to generate and analyze klp-7 null mutants in this species. We therefore cannot exclude the possibility that differences in RNAi efficiency contributed to the observed differences in pronuclear and spindle dynamics, including the relatively mild effect of klp-7(RNAi) in C. inopinata.

Nevertheless, RNAi targeting gpr-1/2, let-99, or klp-7 caused high embryonic lethality in C. inopinata, at levels comparable to those observed in C. elegans. These results indicate that the RNAi treatments produced biologically substantial reductions in gene function, although they do not establish that the extent of depletion was equivalent between the two species. Thus, while differences in RNAi efficiency may have contributed to the observed phenotypic differences, they are unlikely to account fully for all of our findings.

Following the reviewer’s suggestion, we have revised the Results and Discussion sections (Page 14, line 357; Pages 16–17, lines 418–431) to explicitly acknowledge the technical limitations of RNAi analysis in C. inopinata and to qualify our conclusion regarding the differential contributions of KLP-7 and other microtubule regulators in the two species.

Reviewer #2:

Major points 1

Pronuclear and spindle dynamics in both species were assessed at 25�C, which near the upper limit of what C. elegans tolerates and may be near the lower end of what C. inopinata normally experiences. While the rationale for comparing the two species at the same temperature is reasonable, the report would be strengthened by evidence that the observations made for C. inopinata at 25°C are similar to what is seen under culture conditions closer to the preferred temperature for this species.

Response

We thank the reviewer for raising this important point regarding the temperature conditions used for the interspecies comparison. Prior to conducting this experiment, we measured the hatching rates of C. elegans and C. inopinata over a broad temperature range. More than 80% of C. elegans embryos hatched at temperatures between 10°C and 28°C, whereas more than 80% of C. inopinata embryos hatched between 16°C and 32°C. These results indicate that 25°C lies well within the permissive range for embryonic development in both species and is not close to the lower limit for C. inopinata.

Although these hatching data do not directly demonstrate that all aspects of pronuclear and spindle dynamics are identical across the permissive temperature range, they support the conclusion that the dynamics observed at 25°C were examined under physiologically permissive conditions for C. inopinata. Moreover, the use of the same temperature for both species allowed us to compare their embryonic dynamics under equivalent experimental conditions.

To clarify this rationale, we have added the temperature-dependent hatching data to the Supporting Information and described the permissive temperature ranges in the Results section (Page 8, lines 183–184).

Major point 2

The RNAi approaches for C. elegans and C. inopinata are quite different. For C. elegans, L4 larvae were soaked and self-fertilized embryos were analyzed from the resulting adults. For C. inopinata, gravid females were fed RNAi bacteria, and embryos were analyzed from the mated progeny of these females. No comparison of RNAi efficiency, other than embryonic lethality, is provided. Without assessing the degree of depletion, it is difficult to compare the severity of the resulting phenotypes between species. This is particularly important for Cin-klp-7, which shows a weaker phenotype than might be expected. The authors should show that this is not simply due to incomplete depletion. If depletion efficiency cannot be measured directly, the authors should, at minimum, acknowledge this limitation in the Results and Discussion sections.

Response

We agree that differences in the RNAi protocols and efficiencies between the two species are an important limitation, particularly when interpreting the relatively mild Cin-klp-7(RNAi) phenotype. As this concern overlaps with that raised by Reviewer #1, we refer the reviewer to our detailed response above. Briefly, although high embryonic lethality indicates that RNAi substantially reduced gene function, we were unable to directly compare depletion efficiency between the two species and therefore cannot exclude the possibility that incomplete or variable knockdown contributed to the observed phenotypic differences. We have revised the Results and Discussion sections accordingly (Page 14, line 357; Pages 16–17, lines 418–431).

Major point 3

The data presented in Fig. 6 should be quantified similarly to what was done in Fig. 4. While the Cin-gpr-1 RNAi phenotype is clear, the effect of Cin-let-99 depletion is more difficult to assess. This is particularly true for the increased amplitude of spindle oscillations reported by the authors. This difference is not obvious from the data as shown and should be quantified to support the authors’ interpretations.

Response

Following the reviewer’s suggestion, we have quantified the data presented in Fig. 6 and added new panels (Fig. 6C–6E) in the revised manuscript. These newly added panels present the number of rocking cycles, the angular displacement during initial rocking, and the centrosome orientation upon completion of cytokinesis, respectively.

These quantitative data demonstrate that gpr-1/2 RNAi reduced the number of rocking cycles in both species, and let-99 RNAi increased the rocking amplitude in both species. Notably, while klp-7 RNAi reduced the number of rocking cycles in both species, it induced spindle misalignment at the completion of cytokinesis exclusively in C. elegans, but not in C. inopinata.

We have added these new data and descriptions into the Results section (Page 14-15, lines 358–368). In addition, to reflect these quantitative analyses, we have revised the Materials and Methods section under "Quantification of mitotic spindle oscillation" (Page 6, lines 125–131).

Major point 4

In C. inopinata, the NCC forms more posteriorly, then moves more anteriorly, before moving back to its final position at ~60% of A-P axis. Posterior formation of the NCC is attributed to the position of the maternal pronucleus/oocyte nucleus, as well as reduced movement of the male pronucleus. What do the authors think underlies the subsequent over-centration (i.e. increased movement of the NCC to the anterior) in C. inopinata?

Response

We thank the reviewer for this insightful question. We speculate that the excessive anterior displacement of the NCC in C. inopinata reflects a transient imbalance between anterior-directed, microtubule length-dependent pulling forces and cortical pulling forces that normally position and stabilize the NCC near the center of the embryo. Specifically, the anterior-directed forces may initially predominate, causing the NCC to move beyond the center, after which cortical pulling forces reposition it at approximately 60% of the A–P axis. We have added this possible explanation to the Discussion section (Page 17, lines 435–438).

Minor point 1

Lines 189, 217 – 219, 291 – 292, and possibly elsewhere: Add references to original papers for C. elegans observations, e.g. PMID: 8625834 DOI: 10.1242/dev.122.5.1467 should be referenced for oocyte nuclear positioning in C. elegans.

Response

Following the reviewer's suggestion, we have added references to original papers for C. elegans observations throughout the manuscript.

For the oocyte nuclear positioning (Page 9, line 217):

・ Goldstein, B., & Hird, S. N. Specification of the anteroposterior axis in Caenorhabditis elegans. Development 1 May 1996; 122 (5): 1467–1474. doi: https://doi.org/10.1242/dev.122.5.1467

For the NCC and mitotic spindle dynamics (Page 10, Line 338):

・ Albertson, D. G. Formation of the first cleavage spindle in nematode embryos. Developmental Biology, Volume 101, Issue 1, 1984, Pages 61-72, ISSN 0012-1606, doi: https://doi.org/10.1016/0012-1606(84)90117-9.

For the description of the Gα-GPR-LIN-5 complex (Page 13, Line 337):

・ Park, D. H., & Rose, L. S. (2008). Dynamic localization of LIN-5 and GPR-1/2 to cortical force generation domains during spindle positioning. Developmental biology, 315(1), 42-54.

・ Grill, S. W., Gönczy, P., Stelzer, E. H., & Hyman, A. A. (2001). Polarity controls forces governing asymmetric spindle positioning in the Caenorhabditis elegans embryo. Nature, 409(6820), 630-633.

・ Tsou, M. F. B., Hayashi, A., DeBella, L. R., McGrath, G., & Rose, L. S. (2002). LET-99 determines spindle position and is asymmetrically enriched in response to PAR polarity cues in C. elegans embryos.

Minor point 2

Line 62: typo/grammar, remove “the” before “intracellular dynamics”

Response

Following the reviewer's suggestion, we have revised the manuscript accordingly (Page 3, Lines 61). We have also corrected similar grammatical errors elsewhere in the manuscript (Page 3, Lines 62).

Minor point 3

Lines 82-85: Since C. inopinata is a relatively new model, it would be helpful to add technical details regarding culture conditions here, rather than directing readers only to previous work (i.e. ref 14). Both modifications to NGM plates and any differences in propagation due to gonochorism should be added.

Response

Following the reviewer's suggestion, we have added technical details regarding the culture conditions of C. inopinata to the Materials and Methods section (Page 4, Lines 84–89).

Minor point 4

Line 102 – 103: The Methods state that all imaging experiments were performed at 25℃. The authors should indicate how this temperature control was achieved.

Response

Following the reviewer's suggestion, we have added the temperature control method to the Materials and Methods section (Page 5, lines 110–112).

Minor point 5

Line 147: Were antibiotics used in RNAi plates as is typical for RNAi by feeding for C. elegans? If so indicate which antibiotic was used and at what final concentration. If not, it would be helpful to state this and briefly explain the rationale, especially for researchers who may wish to perform RNAi in C. inopinata.

Response

Yes, ampicillin (final concentration, 50 μg/mL) and tetracycline (final concentration, 10 μg/mL) were used in the RNAi plates for C. inopinata. Following the reviewer's suggestion, we have added this information to the Materials and Methods section (Page 7, Lines 160).

Minor point 6

Lines 147 – 151: For readers unfamiliar with C. inopinata, please clarify the RNAi workflow. Were the “gravid adults” used for embryo dissection and imaging mated females, whose parents, both female and male, had been RNAi treated since hatching? Similarly, for embryonic lethality tests, were the females and males used the progeny of the original RNAi-treated “gravid females”?

Response

Following the reviewer’s suggestion, we have clarified the RNAi workflow in the Materials and Methods section (Page 7, lines 161–165). Briefly, untreated adult females (P0) were transferred to RNAi plates and allowed to lay eggs. The F1 males and females were continuously exposed to feeding RNAi from hatching and allowed to mate with each other. The resulting mated F1 females carrying embryos were referred to as “gravid adults.” Their embryos (F2) were used for both live imaging and embryonic lethality assays.

Minor point 7

Line 165: Add to main text that the duration of the first cell cycle was measured as the time between NEBD and the completion of cytokinesis. This is currently only specified in the figure legend.

Response to minor point 7

Following the reviewer’s suggestion, we have revised the main text to define the duration of the first cell division as the interval from nuclear envelope breakdown (NEBD) to the completion of cytokinesis (Page 8, lines 185–186), consistent with the figure legend.

Minor point 8

Line 182 and throughout: Replace “pronucleus” with “pronuclear”, when the word is used as an adjective, as in “pronuclear dynamics”. This terminology/grammar is inconsistent throughout the manuscript.

Response to minor point 8

Following the reviewer's suggestion, we have replaced “pronucleus” with “pronuclear” throughout the manuscript when used adjectivally.

Minor point 9

Lines 190 – 191 and Fig. 2C: Did the authors measure oocyte volume/size? Given the similar size of the embryos, I was surprised that the oocytes and zygote shown in Fig. 2C appear substantially larger in C. inopinata than in C. elegans.

Response

Although we did not directly measure oocyte volume, C. inopinata eggs are significantly larger than those of C. elegans, with an approximately 5.7% greater egg length (Fig. 1B). This difference may reflect a corresponding difference in oocyte size.

We also found that the scale bar in the legend for Fig. 2C was incorrectly labeled as 100 µm rather than 25 µm, which likely contributed to the apparent size discrepancy. We have corrected this error in the revised manuscript (Page 9, line 236).

Minor point 10

Line 278: The phrase “both centrosomes diffused slowly” implies diffusion of intact centrosomes. Consider revising to indicate slower, progressive centrosome disassembly, possibly by diffusion of centrosome components into the cytoplasm.

Response

Following the reviewer’s suggestion, we have revised the Results section by replacing “both centrosomes diffused slowly” with “the centrosome signals gradually decreased,” which more accurately describes the progressive loss of centrosomal fluorescence observed in our imaging (Page 12, line 320).

Minor point 11

Line 285: Add a description to the figure legend for Fig. 5 as to what “1.5 x enhanced” refers to.

Response

We thank the reviewer for pointing this out. The label “1.5 × enhanced” referred to an adjustment of the display range in ImageJ, from 90–700 for C. elegans to 90–500 for C. inopinata, which increased the apparent brightness by approximately 1.5-fold. However, because Fig. 5 shows different transgenes in the two species and is not intended for quantitative comparison of fluorescence intensity, we agreed that this label could be misleading. We have therefore removed “1.5 × enhanced” from Fig. 5 and its legend.

Comments to the Author

3. Have the authors made all data underlying the findings in their manuscript fully available?

The PLOS Data policy requires authors to make all data underlying the findings described in their manuscript fully available without restriction, with rare exception (please refer to the Data Availability Statement in the manuscript PDF file). The data should be provided as part of the manuscript or its supporting information, or deposited to a public repository. For example, in addition to summary statistics, the data points behind means, medians and variance measures should be available. If there are restrictions on publicly sharing data—e.g. participant privacy or use of data from a third party—those must be specified.

Reviewer #2: No

Response

In response to the reviewer’s comment, we have provided all underlying numerical data required to replicate the findings of this study as "Supporting Information" in the public repository "Tohoku University Research Data Lake IZUMI." The corresponding URL has been incorporated into the revised manuscript (Page 23-24, lines 582–604).

Attachment Submitted filename: Response to Reviewers_Oomura.pdf

# Editor report: Decision Letter 1

**DOI:** 10.1371/journal.pone.0352057.r003

**Authors:** Bob Goldstein

24 Sep 2026

Divergence of cortical force-generating mechanisms underlies differences in spindle behavior between *C. elegans*  and *C. inopinata*

PONE-D-26-27765R1

Dear Dr. Sugimoto,

We’re pleased to inform you that your manuscript has been judged scientifically suitable for publication and will be formally accepted for publication once it meets all outstanding technical requirements.

Within one week, you’ll receive an e-mail detailing the required amendments. When these have been addressed, you’ll receive a formal acceptance letter and your manuscript will be scheduled for publication.

An invoice will be generated when your article is formally accepted. Please note, if your institution has a publishing partnership with PLOS and your article meets the relevant criteria, all or part of your publication costs will be covered. Please make sure your user information is up-to-date by logging into Editorial Manager at Editorial Manager® and clicking the ‘Update My Information' link at the top of the page. For questions related to billing, please contact billing support.

If your institution or institutions have a press office, please notify them about your upcoming paper to help maximize its impact. If they’ll be preparing press materials, please inform our press team as soon as possible -- no later than 48 hours after receiving the formal acceptance. Your manuscript will remain under strict press embargo until 2 pm Eastern Time on the date of publication. For more information, please contact onepress@plos.org.

Kind regards,

Bob Goldstein

Academic Editor

PLOS One

# Editor report: Acceptance letter

**DOI:** 10.1371/journal.pone.0352057.r004

**Authors:** Bob Goldstein

PONE-D-26-27765R1

PLOS One

Dear Dr. Sugimoto,

I'm pleased to inform you that your manuscript has been deemed suitable for publication in PLOS One. Congratulations! Your manuscript is now being handed over to our production team.

At this stage, our production department will prepare your paper for publication. This includes ensuring the following:

* All references, tables, and figures are properly cited

* All relevant supporting information is included in the manuscript submission,

* There are no issues that prevent the paper from being properly typeset

You will receive further instructions from the production team, including instructions on how to review your proof when it is ready. Please keep in mind that we are working through a large volume of accepted articles, so please give us a few days to review your paper and let you know the next and final steps.

Lastly, if your institution or institutions have a press office, please let them know about your upcoming paper now to help maximize its impact. If they'll be preparing press materials, please inform our press team within the next 48 hours. Your manuscript will remain under strict press embargo until 2 pm Eastern Time on the date of publication. For more information, please contact onepress@plos.org.

You will receive an invoice from PLOS for your publication fee after your manuscript has reached the completed accept phase. If you receive an email requesting payment before acceptance or for any other service, this may be a phishing scheme. Learn how to identify phishing emails and protect your accounts at https://explore.plos.org/phishing.

If we can help with anything else, please email us at customercare@plos.org.

Thank you for submitting your work to PLOS One and supporting open access.

Kind regards,

PLOS One Editorial Office Staff

on behalf of

Dr. Bob Goldstein

Academic Editor

PLOS One
